# Painful Lytic Lesions of the Foot: A Case Report

**DOI:** 10.5704/MOJ.1503.005

**Published:** 2015-03

**Authors:** R Vaishya, V Vijay, P Ghogare, A Vaish

**Affiliations:** Department of Orthopaedics, Indraprastha Apollo Hospitals, New Delhi, India; * Department of Orthopaedics, Sancheti Institute of Orthopaedics, Pune, India

**Keywords:** Lytic Lesions, Foot, Mycetoma, Histopathology, Infection

## Abstract

The presence of lytic lesions in the bones of foot raises a number of diagnostic possibilities ranging from infection, inflammatory pathology to neoplastic conditions. Although the radiological picture is not pathognomonic of any pathology, clinical history and histopathological examination can help to clinch the diagnosis. We present a case of multiple lytic lesions of the foot and discuss possible differential diagnoses. The patient was diagnosed as a case of madura foot and the lesions responded to surgical debridement and anti-fungal treatment with a good functional outcome. Madura foot is an uncommon, chronic granulomatous fungal or bacterial infection with a predilection in people who walk barefoot. Although known for a specific geographical distribution, madura foot should be kept as a possible diagnosis in patients presenting with lytic lesions of the foot due to population emigration across the world.

## Introduction

Although a large number of bones are present in the foot the lesions in them are infrequently reported in the literature^1^. The patients presenting with radiographic picture of multiple lytic lesions of the foot raise myriad clinical suspicions ranging from benign neoplasms to rapidly progressive infections. Since the symptoms are often non specific, awareness about the various differential diagnoses is essential for the management of these lesions.

## Case Report

A 21 years old male, presented with pain and recurrent nodular swelling over right foot for last four years. He was farmer by occupation. Following the onset of painful swellings, after two years, there was history of a sinus from the foot discharging pus with dark coloured granules. There was history of close contact with an active case of pulmonary tuberculosis (wife). Following the onset of the discharge, the patient visited a local practitioner who sent the pus for culture and sensitivity. The patient was started on oral antibiotics on the basis of sensitivity. The discharge mildly decreased on oral antibiotics but persisted and the patient was advised local dressing. Due to the persistent discharging sinus, patient finally presented to the outpatient department of our hospital.

On local examination, multiple nodular swellings were seen over dorsum, medial and lateral aspects of the mid foot. There was a discharging sinus over the dorsum of the midfoot. The sinus was adherent to the bone and the margins were not undermined. There was diffuse tenderness on the mid and forefoot and the movements of the mid and forefoot were painful and restricted.

**Fig. 1:**
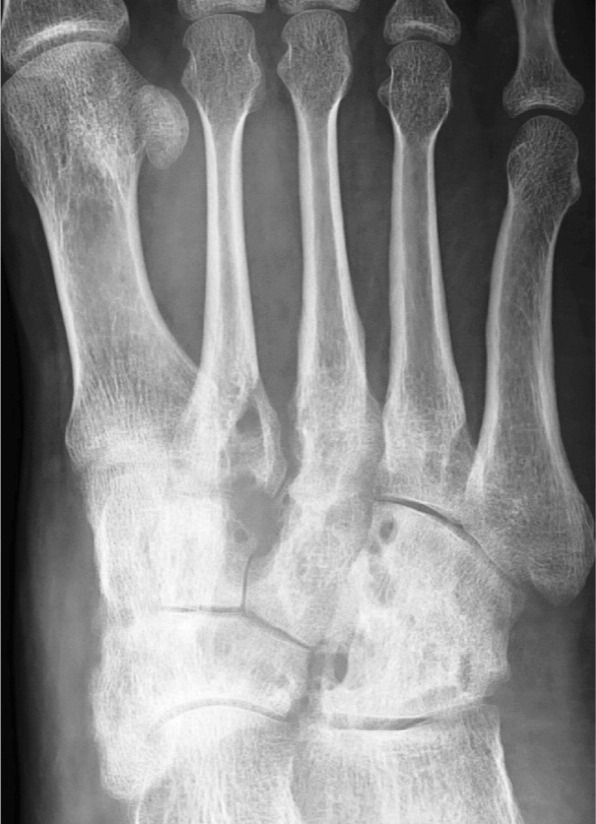
Pre operative radiograph of the foot showing multiple lytic lesions involving the mid foot bones and joints.

Blood investigations revealed marginally high ESR (32) and raised C-reactive protein levels (20ng/dl). Plain radiographs of the foot showed multiple lytic lesions involving the metatarso-cuneiform joints and inter-tarsal joints (Figure 1) and were associated with gross osteoporosis. On the basis of the history and examination, number of diagnostic possibilities arose. Since the patient was a farmer by occupation, the possibility of foreign body prick (like thorn) was considered. Due to the endemic nature and history of close contact with a case of tuberculosis, pedal tuberculosis was also kept as a differential. The other differential diagnoses included rheumatoid, neuropathic and neoplastic involvement of the foot. To rule out the possible differentials and reach the final diagnoses, incision biopsy was planned.

**Fig. 2:**
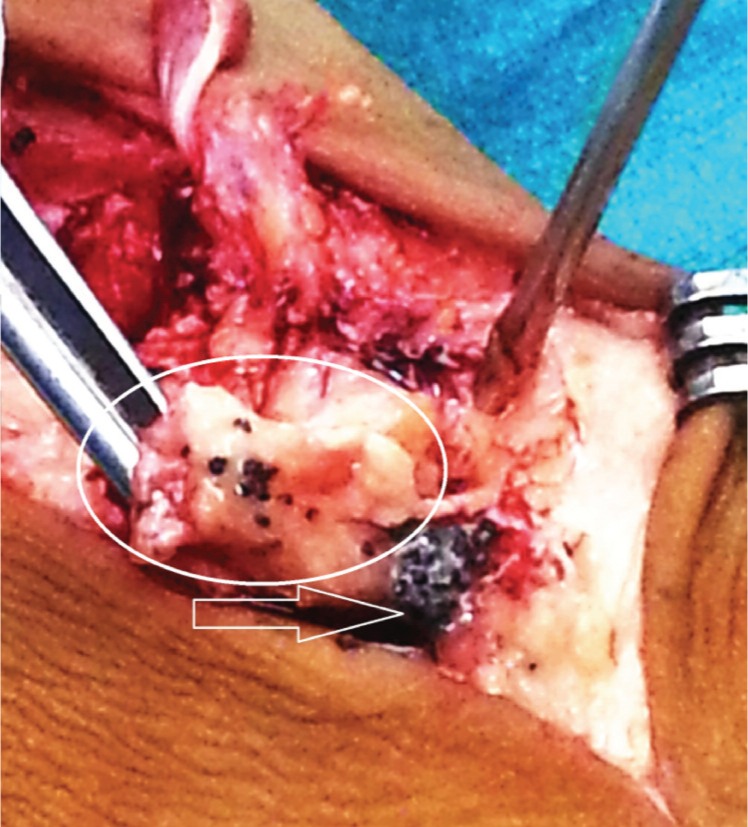
Intra operative photograph showing sequestrum (circle) being held by the forceps and black coloured granules – which represent colonies in the case of madura foot (arrow).

The incisional biopsy was performed from the dorsal aspect of mid foot along with sinus tract excision. The incision revealed pus which was interspersed with dark black coloured granules on gross examination. A big seqeustrum was removed from the tarso-metatarsal junction, with multiple black pigmented granules (Figure 2).

**Fig. 3:**
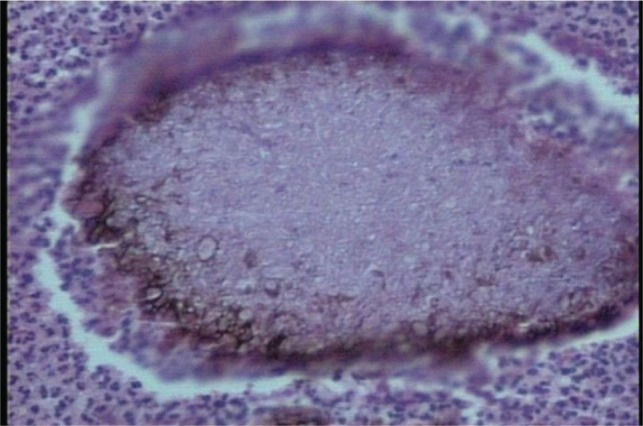
Histopathological picture showing colonies of fungus and coloured granules.

The histo-pathological examination of the tissue revealed colonies comprising of dense tangled meshwork of slender hyphae interspersed with ovoid to round spore like structures. The colonies had brown pigmentation at the periphery (Figure 3). The fungal culture did not grow any organisms.

**Fig. 4:**
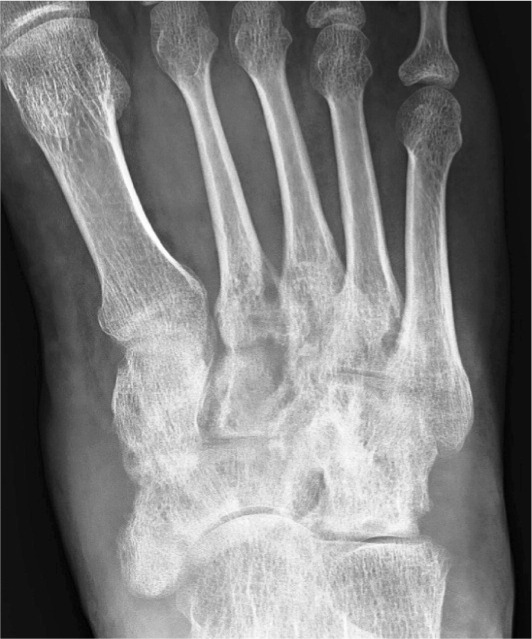
Post operative radiograph of the foot showing healing of the lytic lesions.

On the basis of the histo-pathology findings, the patient was started on oral Itraconazole 200 mg twice a day and Terbinafine 250 mg twice a day for a period of 12 months.

On follow up, the sinus on the foot healed and there was significant improvement in pain. The radiographs did not show any improvement in the lytic lesion in the initial follow ups and the only sign of recovery was the improvement in osteoporosis. The sequential radiographs of the foot revealed resolution in the lytic lesions in the foot as well (Fig 5). The pain improved dramatically. The treatment was finally discontinued after the resolution of the lytic lesions.

## Discussion

The clinical presentation and presence of lytic lesions in the foot as in this case, raised various diagnostic possibilities: infection (tuberculosis, mycetoma), inflammatory (rheumatoid arthritis), neuropathic joints, foreign body injury, primary and metastatic bone tumours, etc.

Bare foot walking invites infections of the foot commonly. This is due to breach in the skin and seeding of bacteria, fungus, etc. Tuberculosis of the foot is rare with an incidence of 0.1-0.3%^2^. It can present as an isolated lytic lesion, synovitis, peri-articular lytic lesion or a diffuse involvement like rheumatoid type of Tuberculosis^2^. Rheumatoid arthritis can present rarely as a diffuse lytic lesion in the mid foot, with gross osteoporosis, a lesion described as “tarsal coalition”^3^.

Foreign body pricks are also common with thorns, wooden splinters and needles etc, especially in people who walk bare foot in the countryside. These injuries may present either as osteolytic or osteoblastic lesions on radiographs and may mimic osteomyelitis, bone tumors and other inflammatory lesions.

Neoplasms of the foot are rare and when they do may present as lytic lesions. The most common benign tumours of the foot are giant cell tumour, enchondroma, chondroblastoma, among others. The foot is also an uncommon site for primary benign neoplasms as described by Uppin *et al*^1^. Metastasis to the foot (acrometastasis) are even rarer.

Mycetoma foot is a chronic, granulomatous infection of the foot, with a waxing and waning course. It mostly affects young males (20-50 years), who are involved in activities involving barefoot walking like in agriculture, farming, etc.^4,5^. Although endemic in parts of Central & South America and Central & East Asia, including India, cases have also been reported from other parts of the world, due to large population emigration^4^.

Mycetoma can be caused by a variety of organisms which include bacteria (Actinomycetoma) and Fungi (Eumycetoma). The most common causative species of Actinomycetoma are Actinomadura, Streptomyces and Nocardia whereas the most common causative fungus is Madurella^4^. The primary infection occurs via micro trauma which is caused by barefoot walking and allows the organism to enter the foot. The incubation period is prolonged and usually asymptomatic, although intermittent diffuse pain has been reported^5^. The classical presentation in the cases of mycetoma consists of tumefaction (tumour-like mass) along with formation of sinus tracts which discharge coloured granules varying from black, yellow and white.

Plain radiographs usually reveal only a soft tissue shadow in the early stages of the disease but involvement of the bones progressively starting from periosteal reaction in a single bone and finally progressing to involve all the foot bones will present as diffuse multiple lytic skeletal lesions^5^. Diffuse osteoporosis distal to the site of the lesion is common due to disuse and compression of distal blood supply by the granuloma 5.

Uppin *et al*^1^ in - a review of 1014 cases of bone lesions in mycetoma, only 52 involved the bones of the hand and feet and of these only two cases of fungal osteomyelitis were observed.

The final diagnosis is usually based on histo-pathology and culture. The differentiation from other infective causes may require positive cultures which are not possible due to the fastidious nature of the fungal organisms and hence treatment may have to be started on the basis of histopathology.

The management of mycetoma foot consists of anti microbial therapy (antifungal and/or anti bacterial) and in resistant cases surgical debridement followed by prolonged course of oral antibiotics, according to the culture sensitivity of the causative organism, usually succeeds. One must be aware that the recurrences of mycetoma are not uncommon, even after an apparent control of the disease process. However, in advanced cases surgical amputation of the involved foot may be required^4,5^.
